# (*E*)-4-Hy­droxy-2-[(2-hy­droxy­phen­yl)iminiometh­yl]phenolate

**DOI:** 10.1107/S1600536810020295

**Published:** 2010-06-05

**Authors:** Naser Eltaher Eltayeb, Siang Guan Teoh, Hoong-Kun Fun, Suchada Chantrapromma

**Affiliations:** a School of Chemical Sciences, Universiti Sains Malaysia, 11800 USM, Penang, Malaysia; b X-ray Crystallography Unit, School of Physics, Universiti Sains Malaysia, 11800 USM, Penang, Malaysia; c Crystal Materials Research Unit, Department of Chemistry, Faculty of Science, Prince of Songkla University, Hat-Yai, Songkhla 90112, Thailand

## Abstract

The title compound, C_13_H_11_NO_3_, crystallizes in a zwitterionic form and has a *trans* configuration about the C=N bond. The mol­ecule is almost planar, the dihedral angle between the two benzene rings being 4.32 (8)°. The two hy­droxy substit­uents are coplanar with each of their attached benzene rings [r.m.s. deviations of 0.0053 (2) and 0.0052 (2) Å]. An intra­molecular N—H⋯O hydrogen bond formed between the iminium N and the phenolate O atom generates an *S*(6) ring motif. In the crystal, the mol­ecules are linked through O—H⋯O hydrogen bonds into chains along [110]. Two neighbouring chains are further connected through O—H⋯O hydrogen bonds in an anti­parallel manner. π–π inter­actions are also observed, with centroid–centroid distances of 3.7115 (19) and 3.743 (2) Å.

## Related literature

For background to Schiff bases and their applications, see: Dao *et al.* (2000 ▶); Kagkelari *et al.* (2009 ▶); Karthikeyan *et al.* (2006 ▶); Sriram *et al.* (2006 ▶). For related structures, see: Eltayeb *et al.* (2009 ▶, 2010*a* ▶,*b* ▶); Tan & Liu (2009 ▶). For the stability of the temperature controller used in the data collection, see Cosier & Glazer, (1986 ▶). For bond-length data, see: Allen *et al.* (1987 ▶). For hydrogen-bond motifs, see: Bernstein *et al.* (1995 ▶).
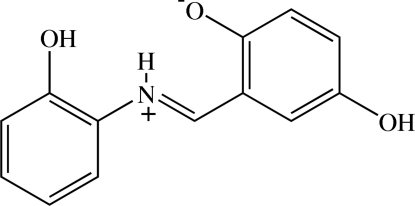


## Experimental

### 

#### Crystal data

C_13_H_11_NO_3_
*M* *_r_* = 229.23
Monoclinic, 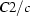

*a* = 11.048 (5) Å
*b* = 8.187 (3) Å
*c* = 22.858 (10) Å
β = 102.242 (13)°
*V* = 2020.5 (15) Å^3^
*Z* = 8
Mo *K*α radiation
μ = 0.11 mm^−1^
*T* = 100 K
0.35 × 0.12 × 0.04 mm

#### Data collection

Bruker APEXII DUO CCD area-detector diffractometer
Absorption correction: multi-scan (*SADABS*; Bruker, 2009 ▶) *T*_min_ = 0.963, *T*_max_ = 0.996
15107 measured reflections
2967 independent reflections
2122 reflections with *I* > 2σ(*I*)
*R*_int_ = 0.087

#### Refinement

*R*[*F* ^2^ > 2σ(*F* ^2^)] = 0.054
*wR*(*F* ^2^) = 0.173
*S* = 1.06
2967 reflections
198 parameters
All H-atom parameters refined
Δρ_max_ = 0.48 e Å^−3^
Δρ_min_ = −0.28 e Å^−3^

### 

Data collection: *APEX2* (Bruker, 2009 ▶); cell refinement: *SAINT* (Bruker, 2009 ▶); data reduction: *SAINT*; program(s) used to solve structure: *SHELXTL* (Sheldrick, 2008 ▶); program(s) used to refine structure: *SHELXTL*; molecular graphics: *SHELXTL*; software used to prepare material for publication: *SHELXTL* and *PLATON* (Spek, 2009 ▶).

## Supplementary Material

Crystal structure: contains datablocks global, I. DOI: 10.1107/S1600536810020295/rz2453sup1.cif

Structure factors: contains datablocks I. DOI: 10.1107/S1600536810020295/rz2453Isup2.hkl

Additional supplementary materials:  crystallographic information; 3D view; checkCIF report

## Figures and Tables

**Table 1:** Hydrogen-bond geometry (Å, °)

*D*—H⋯*A*	*D*—H	H⋯*A*	*D*⋯*A*	*D*—H⋯*A*
O1—H1*O*1⋯O2^i^	0.90 (3)	1.74 (3)	2.625 (2)	166 (3)
O3—H1*O*3⋯O2^ii^	0.95 (3)	1.69 (3)	2.633 (2)	173 (2)
N1—H1*N*1⋯O2	0.89 (3)	1.83 (3)	2.581 (2)	141 (2)
C13—H13⋯O1^ii^	1.00 (2)	2.60 (2)	3.411 (3)	137.8 (18)

Symmetry codes: (i) 
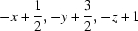
; (ii) 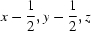.

**Fig. 1.**
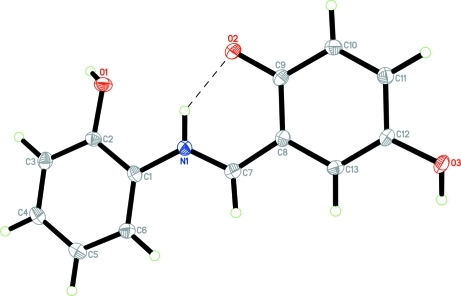
The molecular structure of the title compound, with 50% probability displacement ellipsoids and the atom-numbering scheme. Hydrogen bond is shown as dashed line.

**Fig. 2.**
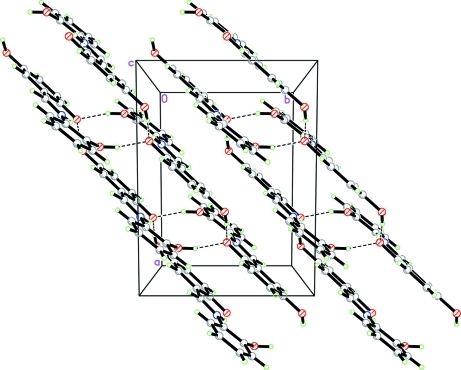
The crystal packing of the title compound viewed down the c axis. Hydrogen bonds are shown as dashed lines.

## supplementary crystallographic information

### Comment

Schiff base ligands and their complexes have varieties of biological activities and applications such as antibacterial and antifungal (Karthikeyan *et al.*, 2006), anticancer (Dao *et al.*, 2000), anti-HIV (Sriram *et al.*, 2006) properties as well as being used in coordination chemistry (Kagkelari *et al.*, 2009). Our on going research on Schiff base ligands and their complexes (Eltayeb *et al.*, 2009; 2010*a*,*b*) has lead us to synthesize the title Schiff base ligand (I) and its crystal structure is reported herein.

The molecule of (I) (Fig. 1), C_13_H_11_NO_3_, crystallizes in a zwitterionic form with cationic iminium and anionic enolate. The molecule exists in a *trans* configuration about the C═N bond [1.302 (2)Å] as indicated by the torsion angle C1–N1–C7–C8 of 178.69 (15)°. The molecule is essentially planar with the dihedral angle of 4.32 (8)° between the two benzene rings. The two hydroxy groups are co-planar with each of their attached benzene rings with the r.m.s. of 0.0053 (2) and 0.0052 (2) Å for the seven non hydrogen atoms of C1–C6/O1 and C8–C13/O3, respectively. An intramolecular N—H···O hydrogen bond (Fig. 1; Table 1) between the NH^+^ with the phenolate O^-^ atom generate an S(6) ring motif (Bernstein *et al.*, 1995) which stabilizes the planarity of the molecule. The bond distances are in normal ranges (Allen *et al.*, 1987) and comparable with those of related structures (Eltayeb *et al.*, 2009; 2010*a*,*b*; Tan & Liu, 2009).

In the crystal packing (Fig. 2), the zwitterionic molecules are linked through O3—H1O3···O2 hydrogen bonds into chains along the [110] direction and two neighboring chains are further connected to each other by O1—H1O1···O2 hydrogen bonds in an antiparallel manner (Table 1). The crystal is stabilized by intermolecular O—H···O hydrogen bonds and weak C—H···O interactions (Table 1). π–π interactions with centroid···centroid distances of Cg_1_···Cg_2_^iii^ = 3.7115 (19) Å and Cg_1_···Cg_2_^iv^ = 3.743 (2) Å were observed (symmetry codes (iii) = -x, 1-y, 1-z and (iv) = 1/2-x, 1/2-y, 1-z); Cg_1_ and Cg_2_ are the centroids of C1–C6 and C8–C13 benzene rings, respectively.

### Experimental

The title compound was synthesized by adding 2,5-dihydroxybenzaldehyde (0.276 g, 2 mmol) to a solution of 2-aminophenol (0.218 g, 2 mmol) in ethanol (30 ml). The mixture was refluxed with stirring for half an hour. The resultant red solution was filtered and the filtrate was evaporated to give a red powder product. Red plate-shaped single crystals of the title compound suitable for *x*-ray structure determination were obtained from acetone by slow evaporation in the refrigerator after a few days.

### Refinement

All H atoms were located from the difference map and isotropically refined. The highest residual electron density peak is located at 0.76 Å from C8 and the deepest hole is located at 1.46 Å from H1O1.

### Crystal data

**Table:**

C_13_H_11_NO_3_	*F*(000) = 960
*M**_r_* = 229.23	*D*_x_ = 1.507 Mg m^−^^3^
Monoclinic, *C*2/*c*	Mo *K*α radiation, λ = 0.71073 Å
Hall symbol: -C 2yc	Cell parameters from 2967 reflections
*a* = 11.048 (5) Å	θ = 1.8–30.1°
*b* = 8.187 (3) Å	µ = 0.11 mm^−^^1^
*c* = 22.858 (10) Å	*T* = 100 K
β = 102.242 (13)°	Plate, red
*V* = 2020.5 (15) Å^3^	0.35 × 0.12 × 0.04 mm
*Z* = 8	

### Data collection

**Table:**

Bruker APEXII DUO CCD area-detector diffractometer	2967 independent reflections
Radiation source: sealed tube	2122 reflections with *I* > 2σ(*I*)
graphite	*R*_int_ = 0.087
φ and ω scans	θ_max_ = 30.1°, θ_min_ = 1.8°
Absorption correction: multi-scan (*SADABS*; Bruker, 2009)	*h* = −15→15
*T*_min_ = 0.963, *T*_max_ = 0.996	*k* = −11→11
15107 measured reflections	*l* = −32→32

### Refinement

**Table:**

Refinement on *F*^2^	Primary atom site location: structure-invariant direct methods
Least-squares matrix: full	Secondary atom site location: difference Fourier map
*R*[*F*^2^ > 2σ(*F*^2^)] = 0.054	Hydrogen site location: inferred from neighbouring sites
*wR*(*F*^2^) = 0.173	All H-atom parameters refined
*S* = 1.06	*w* = 1/[σ^2^(*F*_o_^2^) + (0.1024*P*)^2^ + 0.2914*P*] where *P* = (*F*_o_^2^ + 2*F*_c_^2^)/3
2967 reflections	(Δ/σ)_max_ = 0.001
198 parameters	Δρ_max_ = 0.48 e Å^−^^3^
0 restraints	Δρ_min_ = −0.28 e Å^−^^3^

### Special details

**Table:**

Experimental. The crystal was placed in the cold stream of an Oxford Cryosystems Cobra open-flow nitrogen cryostat (Cosier & Glazer, 1986) operating at 100.0 (1) K.
Geometry. All esds (except the esd in the dihedral angle between two l.s. planes) are estimated using the full covariance matrix. The cell esds are taken into account individually in the estimation of esds in distances, angles and torsion angles; correlations between esds in cell parameters are only used when they are defined by crystal symmetry. An approximate (isotropic) treatment of cell esds is used for estimating esds involving l.s. planes.
Refinement. Refinement of F^2^ against ALL reflections. The weighted R-factor wR and goodness of fit S are based on F^2^, conventional R-factors R are based on F, with F set to zero for negative F^2^. The threshold expression of F^2^ > 2sigma(F^2^) is used only for calculating R-factors(gt) etc. and is not relevant to the choice of reflections for refinement. R-factors based on F^2^ are statistically about twice as large as those based on F, and R- factors based on ALL data will be even larger.

### Fractional atomic coordinates and isotropic or equivalent isotropic displacement parameters (Å^2^)

**Table:**

	*x*	*y*	*z*	*U*_iso_*/*U*_eq_	
O1	0.33647 (12)	0.68246 (15)	0.56400 (5)	0.0181 (3)	
H1O1	0.339 (3)	0.792 (4)	0.5686 (14)	0.049 (8)*	
O2	0.19184 (11)	0.49929 (14)	0.43502 (5)	0.0172 (3)	
O3	−0.15299 (12)	0.01393 (15)	0.36222 (6)	0.0195 (3)	
H1O3	−0.212 (3)	0.001 (3)	0.3867 (14)	0.045 (8)*	
N1	0.18126 (13)	0.43402 (17)	0.54431 (6)	0.0136 (3)	
H1N1	0.213 (2)	0.484 (3)	0.5165 (12)	0.034 (7)*	
C1	0.22799 (14)	0.47819 (19)	0.60450 (7)	0.0137 (3)	
C2	0.30862 (15)	0.6115 (2)	0.61334 (7)	0.0147 (3)	
C3	0.35926 (16)	0.6624 (2)	0.67122 (8)	0.0167 (3)	
H3	0.413 (2)	0.755 (3)	0.6766 (10)	0.024 (6)*	
C4	0.32876 (16)	0.5824 (2)	0.71942 (8)	0.0177 (3)	
H4	0.363 (2)	0.614 (3)	0.7600 (11)	0.026 (6)*	
C5	0.24766 (16)	0.4510 (2)	0.71042 (8)	0.0178 (3)	
H5	0.227 (2)	0.393 (3)	0.7447 (11)	0.028 (6)*	
C6	0.19743 (16)	0.3972 (2)	0.65277 (7)	0.0161 (3)	
H6	0.1437 (19)	0.309 (3)	0.6478 (9)	0.016 (5)*	
C7	0.09838 (15)	0.32381 (19)	0.52344 (7)	0.0142 (3)	
H7	0.0643 (18)	0.254 (2)	0.5512 (9)	0.013 (5)*	
C8	0.05807 (15)	0.29461 (19)	0.46077 (7)	0.0139 (3)	
C9	0.10800 (15)	0.38498 (19)	0.41820 (7)	0.0139 (3)	
C10	0.06509 (16)	0.3443 (2)	0.35726 (7)	0.0160 (3)	
H10	0.101 (2)	0.405 (3)	0.3290 (11)	0.027 (6)*	
C11	−0.02034 (15)	0.2222 (2)	0.34016 (7)	0.0156 (3)	
H11	−0.049 (2)	0.192 (3)	0.2956 (11)	0.025 (6)*	
C12	−0.06925 (15)	0.1342 (2)	0.38267 (7)	0.0144 (3)	
C13	−0.03095 (15)	0.17081 (19)	0.44220 (7)	0.0146 (3)	
H13	−0.066 (2)	0.111 (3)	0.4732 (10)	0.024 (6)*	

### Atomic displacement parameters (Å^2^)

**Table:**

	*U*^11^	*U*^22^	*U*^33^	*U*^12^	*U*^13^	*U*^23^
O1	0.0240 (7)	0.0158 (6)	0.0154 (6)	−0.0033 (5)	0.0063 (5)	0.0012 (4)
O2	0.0194 (6)	0.0153 (6)	0.0168 (6)	−0.0061 (5)	0.0040 (5)	−0.0009 (4)
O3	0.0194 (6)	0.0202 (6)	0.0198 (6)	−0.0099 (5)	0.0062 (5)	−0.0055 (5)
N1	0.0137 (6)	0.0136 (6)	0.0131 (6)	−0.0001 (5)	0.0018 (5)	0.0008 (5)
C1	0.0141 (7)	0.0136 (7)	0.0128 (7)	0.0030 (6)	0.0014 (6)	−0.0005 (5)
C2	0.0157 (7)	0.0140 (7)	0.0146 (7)	0.0013 (6)	0.0037 (6)	0.0021 (6)
C3	0.0169 (8)	0.0139 (7)	0.0182 (8)	−0.0009 (6)	0.0014 (6)	−0.0019 (6)
C4	0.0205 (8)	0.0174 (8)	0.0139 (7)	0.0026 (6)	0.0011 (6)	−0.0008 (6)
C5	0.0203 (8)	0.0185 (8)	0.0150 (7)	0.0031 (7)	0.0045 (6)	0.0021 (6)
C6	0.0168 (8)	0.0151 (8)	0.0163 (8)	−0.0005 (6)	0.0036 (6)	0.0016 (6)
C7	0.0134 (7)	0.0131 (7)	0.0164 (7)	0.0011 (6)	0.0037 (6)	0.0015 (6)
C8	0.0133 (7)	0.0130 (7)	0.0152 (7)	−0.0002 (6)	0.0025 (6)	−0.0010 (6)
C9	0.0119 (7)	0.0127 (7)	0.0172 (7)	0.0002 (6)	0.0032 (6)	−0.0001 (6)
C10	0.0176 (8)	0.0159 (7)	0.0153 (7)	−0.0014 (6)	0.0049 (6)	0.0010 (6)
C11	0.0143 (7)	0.0175 (7)	0.0151 (7)	0.0004 (6)	0.0035 (6)	−0.0011 (6)
C12	0.0125 (7)	0.0123 (7)	0.0183 (8)	−0.0021 (6)	0.0031 (6)	−0.0016 (6)
C13	0.0155 (7)	0.0134 (7)	0.0158 (7)	−0.0012 (6)	0.0052 (6)	0.0010 (6)

### Geometric parameters (Å, °)

**Table:**

O1—C2	1.3608 (19)	C5—C6	1.389 (2)
O1—H1O1	0.91 (3)	C5—H5	0.98 (2)
O2—C9	1.316 (2)	C6—H6	0.93 (2)
O3—C12	1.364 (2)	C7—C8	1.427 (2)
O3—H1O3	0.95 (3)	C7—H7	0.99 (2)
N1—C7	1.302 (2)	C8—C13	1.413 (2)
N1—C1	1.410 (2)	C8—C9	1.423 (2)
N1—H1N1	0.89 (3)	C9—C10	1.413 (2)
C1—C6	1.389 (2)	C10—C11	1.373 (2)
C1—C2	1.396 (2)	C10—H10	0.97 (2)
C2—C3	1.387 (2)	C11—C12	1.406 (2)
C3—C4	1.383 (2)	C11—H11	1.03 (2)
C3—H3	0.96 (2)	C12—C13	1.370 (2)
C4—C5	1.387 (3)	C13—H13	1.00 (2)
C4—H4	0.96 (2)		
			
C2—O1—H1O1	109.4 (19)	C1—C6—H6	122.1 (13)
C12—O3—H1O3	112.0 (17)	N1—C7—C8	121.96 (15)
C7—N1—C1	128.22 (15)	N1—C7—H7	120.0 (12)
C7—N1—H1N1	114.2 (17)	C8—C7—H7	118.0 (12)
C1—N1—H1N1	117.6 (17)	C13—C8—C9	120.89 (14)
C6—C1—C2	120.88 (14)	C13—C8—C7	118.07 (14)
C6—C1—N1	123.52 (15)	C9—C8—C7	121.02 (15)
C2—C1—N1	115.60 (14)	O2—C9—C10	121.61 (14)
O1—C2—C3	123.09 (15)	O2—C9—C8	121.39 (14)
O1—C2—C1	117.61 (14)	C10—C9—C8	116.99 (15)
C3—C2—C1	119.28 (15)	C11—C10—C9	121.25 (15)
C4—C3—C2	120.03 (16)	C11—C10—H10	122.8 (14)
C4—C3—H3	121.6 (14)	C9—C10—H10	116.0 (14)
C2—C3—H3	118.3 (14)	C10—C11—C12	121.19 (15)
C3—C4—C5	120.51 (15)	C10—C11—H11	120.5 (12)
C3—C4—H4	122.0 (14)	C12—C11—H11	118.3 (12)
C5—C4—H4	117.5 (14)	O3—C12—C13	122.86 (14)
C4—C5—C6	120.17 (16)	O3—C12—C11	117.72 (15)
C4—C5—H5	120.3 (14)	C13—C12—C11	119.42 (15)
C6—C5—H5	119.5 (14)	C12—C13—C8	120.25 (14)
C5—C6—C1	119.12 (16)	C12—C13—H13	120.8 (13)
C5—C6—H6	118.8 (13)	C8—C13—H13	119.0 (13)
			
C7—N1—C1—C6	5.5 (3)	N1—C7—C8—C9	0.4 (2)
C7—N1—C1—C2	−174.41 (15)	C13—C8—C9—O2	−179.28 (14)
C6—C1—C2—O1	178.56 (15)	C7—C8—C9—O2	−0.7 (2)
N1—C1—C2—O1	−1.6 (2)	C13—C8—C9—C10	−0.4 (2)
C6—C1—C2—C3	0.5 (2)	C7—C8—C9—C10	178.20 (15)
N1—C1—C2—C3	−179.68 (14)	O2—C9—C10—C11	178.48 (15)
O1—C2—C3—C4	−178.66 (15)	C8—C9—C10—C11	−0.4 (2)
C1—C2—C3—C4	−0.7 (2)	C9—C10—C11—C12	0.6 (3)
C2—C3—C4—C5	0.0 (3)	C10—C11—C12—O3	−179.51 (15)
C3—C4—C5—C6	0.8 (3)	C10—C11—C12—C13	0.0 (2)
C4—C5—C6—C1	−1.0 (3)	O3—C12—C13—C8	178.68 (14)
C2—C1—C6—C5	0.4 (2)	C11—C12—C13—C8	−0.8 (2)
N1—C1—C6—C5	−179.48 (15)	C9—C8—C13—C12	1.0 (2)
C1—N1—C7—C8	178.69 (15)	C7—C8—C13—C12	−177.65 (15)
N1—C7—C8—C13	179.06 (14)		

### Hydrogen-bond geometry (Å, °)

**Table:**

*D*—H···*A*	*D*—H	H···*A*	*D*···*A*	*D*—H···*A*
O1—H1O1···O2^i^	0.90 (3)	1.74 (3)	2.625 (2)	166 (3)
O3—H1O3···O2^ii^	0.95 (3)	1.69 (3)	2.633 (2)	173 (2)
N1—H1N1···O2	0.89 (3)	1.83 (3)	2.581 (2)	141 (2)
C13—H13···O1^ii^	1.00 (2)	2.60 (2)	3.411 (3)	137.8 (18)

Symmetry codes: (i) −*x*+1/2, −*y*+3/2, −*z*+1; (ii) *x*−1/2, *y*−1/2, *z*.
